# Gestational diabetes mellitus: Impacts on fetal neurodevelopment, gut dysbiosis, and the promise of precision medicine

**DOI:** 10.3389/fmolb.2024.1420664

**Published:** 2024-07-11

**Authors:** Michelle Biete, Sona Vasudevan

**Affiliations:** Department of Biochemistry, Molecular and Cellular Biology, Georgetown University Medical Center, Washington, DC, United States

**Keywords:** gut–brain axis, metabolic programming, systems medicine, omics, maternal microbiome, short-chain fatty acid

## Abstract

Gestational diabetes mellitus (GDM) is a common metabolic disorder affecting approximately 16.5% of pregnancies worldwide and causing significant health concerns. GDM is a serious pregnancy complication caused by chronic insulin resistance in the mother and has been associated with the development of neurodevelopmental disorders in offspring. Emerging data support the notion that GDM affects both the maternal and fetal microbiome, altering the composition and function of the gut microbiota, resulting in dysbiosis. The observed dysregulation of microbial presence in GDM pregnancies has been connected to fetal neurodevelopmental problems. Several reviews have focused on the intricate development of maternal dysbiosis affecting the fetal microbiome. Omics data have been instrumental in deciphering the underlying relationship among GDM, gut dysbiosis, and fetal neurodevelopment, paving the way for precision medicine. Microbiome-associated omics analyses help elucidate how dysbiosis contributes to metabolic disturbances and inflammation, linking microbial changes to adverse pregnancy outcomes such as those seen in GDM. Integrating omics data across these different layers—genomics, transcriptomics, proteomics, metabolomics, and microbiomics—offers a comprehensive view of the molecular landscape underlying GDM. This review outlines the affected pathways and proposes future developments and possible personalized therapeutic interventions by integrating omics data on the maternal microbiome, genetics, lifestyle factors, and other relevant biomarkers aimed at identifying women at high risk of developing GDM. For example, machine learning tools have emerged with powerful capabilities to extract meaningful insights from large datasets.

## Introduction

The advent of high-throughput sequencing, along with large-scale initiatives such as the Human Microbiome Project (Wu et al., 2024), has paved the way for studies aimed at understanding microbial networks, microbiome–disease associations, and the close connection between host–microbiota interactions. The microbiota forms intricate ecosystems with the host and is uniquely adapted to the constantly fluctuating physiology of the host. A huge body of evidence shows how microbiome imbalance, referred to as microbiome dysbiosis, affects the maternal metabolic profile, contributing to pregnancy complications leading to compromised neonatal health (Dunlop et al., 2015). Gestational diabetes mellitus (GDM) is a serious pregnancy complication resulting in chronic insulin resistance in the mother and affecting approximately 16.5% of pregnancies worldwide (Plows et al., 2018). In GDM, mothers develop abnormal glucose intolerance, which is diagnosed between 24 and 28 weeks of gestation when the oral glucose tolerance test is carried out (Wang et al., 2018). As a result of this glucose intolerance, maternal blood has a higher concentration of glucose than that in healthy mothers, leading to maternal metabolic morbidity, including diabetes, which is generally referred to as GDM.

Recent research on large maternal-child cohort studies has shown a consistent association of GDM with offspring neurodevelopmental disorders, particularly attention-deficit/hyperactivity, schizophrenia, intellectual disability, anxiety, and depression (Shook et al., 2020; Chen et al., 2020a; Edlow, 2021; Rodolaki et al., 2023). With diabetes in women of reproductive age increasing, it is anticipated that rates of neurodevelopment and psychiatric morbidity in children and young adults will be on the verge of a public health crisis (Edlow, 2021).

Emerging evidence also suggests the crucial role of the gut microbiota and its metabolites in the regulation of neurodevelopment and cognition via the gut–brain axis (Iannone et al., 2019; Morais et al., 2020; Grochowska et al., 2019; Ullah et al., 2023). In GDM, maternal microbiome dysbiosis has been attributed to infants developing obesity, long-term neurological defects, and gastrointestinal and metabolic diseases later on in their lives (Su et al., 2018).

This review focuses on the current understanding of the metabolomic implications of maternal dysbiosis on fetal neurodevelopment and will discuss possible mechanisms that are deregulated in GDM

## GDM and its effects on fetal neurodevelopment

Colonization of the human gut microbiota starts during pregnancy. It co-occurs with brain development through the maternal-fetal *in utero* translocation of gut microbiota, which was confirmed by studies using a mouse model (Younge et al., 2019; Dash et al., 2022). The gut microbiome is crucial for fetal development, especially neuronal plus glial development and maturation (Dash et al., 2022). Having dysbiosis can lead to dysregulated immune activation, causing systemic inflammation and leading to neurodevelopmental psychiatric disorders caused by atypical brain development (Dash et al., 2022). A comparative study between germ-free and specific-pathogen-free mice showed that gut microbial metabolites can cross through the placenta to the fetal compartment, showing gut microbes’ ability to affect fetal development (Pessa-Morikawa et al., 2022). A microbial component important for neurodevelopment includes peptidoglycan (PG), a bacterial cell wall that activates TLR2 to trigger an increase in FOXG1 expression, which regulates neurogenesis and induces neuronal proliferation in the forebrain area (Dash et al., 2022). Other microbial metabolites that gut microbes release into the gut lumen known to promote neurogenesis include the neurotransmitter serotonin and short-chain fatty acids (SCFAs) such as butyrate, acetate, and propionate (Agnihotri and Moharjeri, 2022; Dash et al., 2022). SCFAs have been shown to act upon the vagal, immune, and endocrine pathways, with studies showing an increased growth rate and expression of proliferation-related genes in human neural progenitor cells when exposed to physiologically relevant SCFA concentrations (Agnihotri and Moharjeri, 2022). Additionally, common brain diseases were shown to have reduced SCFAs; these include autism spectrum disorder, attention deficit hyperactivity, schizophrenia, and major depressive disorder (Agnihotri and Moharjeri, 2022). SCFAs have been shown to influence gap junction gating in the gut and blood–brain barrier integrity, contributing to brain homeostasis, modulating neuroinflammation, and stimulating brain-derived neurotrophic factor (BDNF) expression. Yang et al. (2019) showed that SCFA treatment of human neural progenitor cells at physiological levels influenced the expression of the neurogenesis, proliferation, and apoptosis-related genes ATR, BCL2, BID, CASP8, CKD2, E2F1, FAS, NDN, and VEGFA (Agnihotri and Moharjeri, 2022; Frerichs et al., 2024). BDNF is a crucial neurotrophin for neuronal differentiation, plasticity, and the establishment of synaptogenesis (Rodolaki et al., 2023). Decreased BDNF expression has been associated with neuronal loss, such as Parkinson’s and Alzheimer’s disease and autism spectrum disorders (Rodolaki et al., 2023).

## Inflammation and early signs of GDM

Possible mechanisms that are deregulated in GDM that could negatively affect fetal neurodevelopment include inflammation and deficiency in maternal microbial metabolites and docosahexaenoic acid (DHA) delivery to the fetus (Mishra et al., 2020; Edlow, 2021; Rodolaski et al., 2023; Sajdel-Sulkowska, 2023). Pinto et al. (2023) found that women in their first trimester of pregnancy exhibit elevated levels of serum pro-inflammatory cytokines such as interleukins (IL-4, IL-6, and IL-8), granulocyte-macrophage colony-stimulating factor, and tumor necrosis factor-alpha way before the onset of GDM. Additionally, the study found that this heightened inflammation is initiated by microbial dysbiosis, leading to the induction of GDM during the third trimester (Pinto et al., 2023). In particular, the study found that the bacteria *Prevotella*, which plays an important role in maintaining glucose homeostasis in the host, was found to be under-represented in women who developed GDM.

In addition, the elevated pro-inflammatory cytokines have been associated with the development of insulin resistance, further strengthening the role of the gut microbiome in GDM (Esser et al., 2014). Both the maternal and fetal microbiome play a role in the development of neonates, and therefore, the observed dysregulation of microbial presence in GDM pregnancies could result in fetal neurodevelopmental problems, as several studies have linked diabetes during gestation to future psychiatric diseases in offspring (Comte et al., 2020; Rodolaski et al., 2023).

## Importance of LC-PUFAs and SCFAs in fetal neurodevelopment

DHA is an essential omega-3 long-chain polyunsaturated fatty acid (LC-PUFA) required for fetal neurodevelopment *in utero* (Basak et al., 2020). In humans, DHA makes up 15% of all the fatty acids in the frontal cortex and plays a role in brain development and the development of the placenta (Basak et al., 2020). Based on the trimester, DHA plays a specific role in developing the fetoplacental unit (Basak et al., 2020). During the second trimester, DHA and its metabolites help regulate immunocompetence by maintaining oxidative stress, combating elevated reactive oxygen species, and maintaining a pro- and anti-inflammatory balance between the maternal and fetal interface to improve maternal-fetal immunocompetence (Basak et al., 2020; Rodolaki et al., 2023). The third trimester is when DHA is delivered to the fetal brain via the placenta, playing a role in several brain development processes such as neurogenesis, synaptogenesis, brain plasticity, inflammatory signaling, and neuroprotection (Basak et al., 2020). DHA also plays a role in maintaining normal neurotrophin levels such as BDNF, which, when deficient, will disturb neurotrophin regulation, such as in diabetic pregnancies (Rodolaki et al., 2023).

Additionally, LC-PUFAs have a positive effect in maintaining a healthy microbiome by promoting the expression of anti-inflammatory compounds such as SCFAs, which have been shown to influence the gut–brain axis (Ahmed et al., 2022). Butyric acid, an SCFA, may function to regulate the production of neurotrophic factors such as BDNF, which conducts signals through the vagal nerve to the brain to promote the production of neurotransmitters in the central nervous system (CNS) (Ahmed et al., 2022). Microbial species such as *Ruminococcaceae*, *Lachnospiraceae*, *Bacteroidetes*, and *Akkermansia muciniphila* are the main producers of SCFAs such as butyrate, propionate, and acetate (Andrioaie et al., 2022)*.* SCFAs have also been shown to regulate the early development of the neural system as they affect the growth rate of human neural progenitor cells generated from embryonic stem cells (Yang et al., 2019).

## Mechanisms of the microbiota–gut–brain axis in GDM

The microbiota–gut–brain axis has been well connected to neurodevelopmental and neuropsychiatric disorders due to multiple pathways through which microbial metabolites are involved in the body, including the connection with the production of cytokines and neurotransmitters (Warner, 2018; McGuinness et al., 2022). An imbalance of microbial metabolites in the gut has also been linked to inflammation, resulting in neurodevelopmental and behavioral disorders. A study conducted in maternal-separated rats by Pusceddu et al. (2015) showed that administering EPA/DHA restored the gut microbiome’s *Firmicutes/Bacteroidetes* ratio in these rats. As a result, this combination improved their inflammatory condition, increasing the abundance of species that produce SCFAs, such as butyrate, and decreasing the number of lactic acid bacteria (Pusceddu et al., 2015)*.* This shows the strong connection between the gut–brain axis and how the long-term administration of DHA/EPA influenced the gut microbiota composition, helping to restore the normal functionality of the gut–brain axis (Costantini et al., 2017).

## Fetal microbiome–gut–brain axis: Maternal gut dysbiosis composition in GDM

Microbial dysbiosis has been found in the maternal gut microbiome of GDM pregnancies. The intestinal microbiota has been shown to modulate insulin resistance and the body’s inflammatory response. By understanding its composition, we may be able to develop preventive strategies and treatments for GDM (Neri et al., 2021). The GDM gut microbiomes have been shown to have a higher *Firmicutes/Bacteroidetes* ratio (Neri et al., 2021; Silas et al., 2021); a higher relative abundance of *Bacteroides* (Chen et al., 2020b; Sililas et al., 2021; Su et al., 2021), *Ruminococcus* (Li et al., 2021; Mora-Janiszewska et al., 2021), *Roseburia* (Li et al., 2021; Mora-Janiszewska et al., 2021), *Lachnospiraceae* (Cortez et al., 2018; Ma et al., 2020; Neri et al., 2021), *Phascolarctobacterium* (Cortez et al., 2018; Neri et al., 2021), *Akkermansia* (Cortez et al., 2018; Eicher and Mohajeri, 2022), and *Christensenella* (Cortez et al., 2018; Neri et al., 2021); and a decrease in *Eubacterium* (Jiang et al., 2018; Ye et al., 2019; Festa et al., 2020; Neri et al., 2021), *Lactobacillus* (Jiang et al., 2015; Ponzo et al., 2019; Festa et al., 2020; Dicks et al., 2021; Neri et al., 2021; Eicher and Mohajeri, 2022), and *Prevotella* (Janssens et al., 2018; Ponzo et al., 2019; Festa et al., 2020; Neri et al., 2021; Eicher and Mohajeri, 2022; Wishart et al., 2022; Pinto et al., 2023), making these species possible markers*. Coprococcus* was found to be decreased in abundance before 20 weeks of gestation, showing that it may play a role in the development of GDM as it was previously shown to be positively correlated with a maternal gastric inhibitory peptide that stimulates the production of insulin (Neri et al., 2021; Vuong, 2022). *Coprococcus* is also a butyrate-producing bacterium which is an SCFA associated with neural function (Neri et al., 2021). Two studies conducted by Bassols et al. (2016) and Ferrocino et al. (2018) further showed that there was a decreased prevalence of *Acinetobacter* in the maternal microbiome*,* which is associated with a more adverse maternal inflammatory and metabolic phenotype, signifying its potential to be a GDM biomarker (Bassols et al., 2016; Ferrocino et al., 2018; Mora-Janiszewska et al., 2022). As the gut microbiome changes throughout pregnancy, it affects the maternal metabolism and fetal development. Table 1 summarizes the effects of the different microbes in GDM.

**TABLE 1:** Maternal and fetal dysbiosis of gut microbiome in women with GDM.

Sample type	Increased abundance	Potential effects on microbial metabolites	Reference
Maternal gut microbiome	*Firmicutes/Bacteroidetes* ratio	Ratio is impacted by the presence of DHA that helps regulate the abundance of bacterial species that produce SCFAs	Neri et al. (2021) and Silas et al. (2021)
Maternal gut microbiome	*Ruminococcus*	SCFA producer	Li et al. (2021) and Mora-Janiszewska et al. (2022)
Maternal gut microbiome	*Roseburia*	Increased levels have been associated with major depressive disorder	Jiang et al. (2015), Li et al. (2021), and Mora-Janiszewska et al. (2022)
Maternal gut microbiome	*Lachnospiraceae*	SCFA producer and found in dysbiotic conditions such as in women with GDM.	Cortez et al. (2018), Ma et al. (2020), and Neri et al. (2021)
Maternal gut microbiome	*Phascolarctobacterium*	Found in dysbiotic conditions such as in women with GDM.	Cortez et al. (2018) and Neri et al. (2021)
Maternal gut microbiome	*Christensenella*	Found in dysbiotic conditions such as in women with GDM.	Cortez et al. (2018) and Neri et al. (2021)
Maternal gut microbiome	*Akkermansia*	SCFA producer and plays a systematic role in controlling metabolic syndromes	Cortez et al. (2018) and Eicher and Mohajeri (2022)
Infant gut microbiota	*Escherichia*	Pro-inflammatory taxa and serotonin producer	Ponzo et al. (2019) and Roth et al. (2021)
Infant gut microbiota	*Parabacteroides*	Pro-inflammatory taxa. Increased levels have been associated with major depressive disorder	Jiang et al. (2015), Janssens et al. (2018), Ponzo et al. (2019), and Wishart et al. (2022)
Maternal gut microbiome	*Akkermansia*	SCFA producer and plays a systematic role in controlling metabolic syndromes	Cortez et al. (2018) and Neri et al. (2021)
Maternal gut microbiome	*Eubacterium*	Decreased levels have been associated with generalized anxiety disorder	Jiang et al. (2018), Ye et al. (2019), Festa et al. (2020), and Neri et al. (2021)
Maternal gut microbiome	*Lactobacillus*	Anti-inflammatory serotonin and GABA producer	Jiang et al. (2015), Ponzo et al. (2019), Festa et al. (2020), Dicks et al. (2021), Neri et al. (2021), and Eicher and Mohajeri (2022)
Maternal gut microbiome	*Prevotella*	Anti-inflammatory and vitamin B1 producer. The microbial metabolites help modulate dopamine and serotonin. Decreased levels have been associated with major depressive disorder	Janssens et al. (2018), Ponzo et al. (2019), Festa et al. (2020), Neri et al. (2021), Eicher and Mohajeri (2022), Wishart et al. (2022), and Pinto et al. (2023)
Maternal gut microbiome	*Acinetobacter*	Associated with adverse maternal inflammatory and metabolic phenotype	Bassols et al. (2016) and Ferrocino et al. (2018)
Maternal gut microbiome	*Coprococcus*	Decreased abundance before 20 weeks of gestation. It produces the SCFA, butyrate	Neri et al. (2021)
Newborn meconium microbiome	*Prevotella*	May play a role in metabolic characteristics. The absence of *Prevotella* may cause the gastrointestinal disease associated with autism. Decreased levels of *Prevotella* have also been associated with anxiety and depression	Kang et al. (2013), Jiang et al. (2015), Ponzo et al. (2019), Dicks et al. (2021), and Li et al. (2021)
Newborn meconium microbiome	*Bacteroides*	May play a role in metabolic characteristics. Decreased levels of *Bacteroides* have also been associated with anxiety and depression	Jiang et al. (2015), Ponzo et al. (2019), Dicks et al. (2021), and Li et al. (2021)
Newborn meconium microbiome	*Lactobacillus*	May play a role in metabolic characteristics. Decreased levels of *Lactobacillus* have also been associated with anxiety and depression	Aizawa et al. (2016), Ponzo et al. (2019), and Li et al. (2021)

GDM has been associated with impaired DHA materno-fetal transfer with lower levels of LC-PUFAs found in placental and fetal compartments, whereas DHA levels were 11%–44% higher in GDM patients than in non-diabetic pregnancies (Pagán et al., 2013; Mishra et al., 2020). One possible mechanism for this significantly decreased DHA transfer is the insulin resistance in GDM, which exposes trophoblasts responsible for placental DHA transfer to high glucose levels, hindering their function (Mishra et al., 2020). As a result, the lack of availability of DHA could hinder the plasticity and maturation of the fetal brain, especially in the third trimester, and cause dysregulation of the fetal microbiome that could affect brain function throughout adulthood (Basak et al., 2022). The dysbiosis of the fetal microbiome in GDM pregnancies has also been observed by Wang et al. (2018), which could affect the production of DHA and LC-PUFAs by the fetal intestines, further impacting neuronal development (Bernhard et al., 2014).

## Effects of GDM on gut microbiota-derived metabolites in fetal neurodevelopment

**FIGURE 1:**
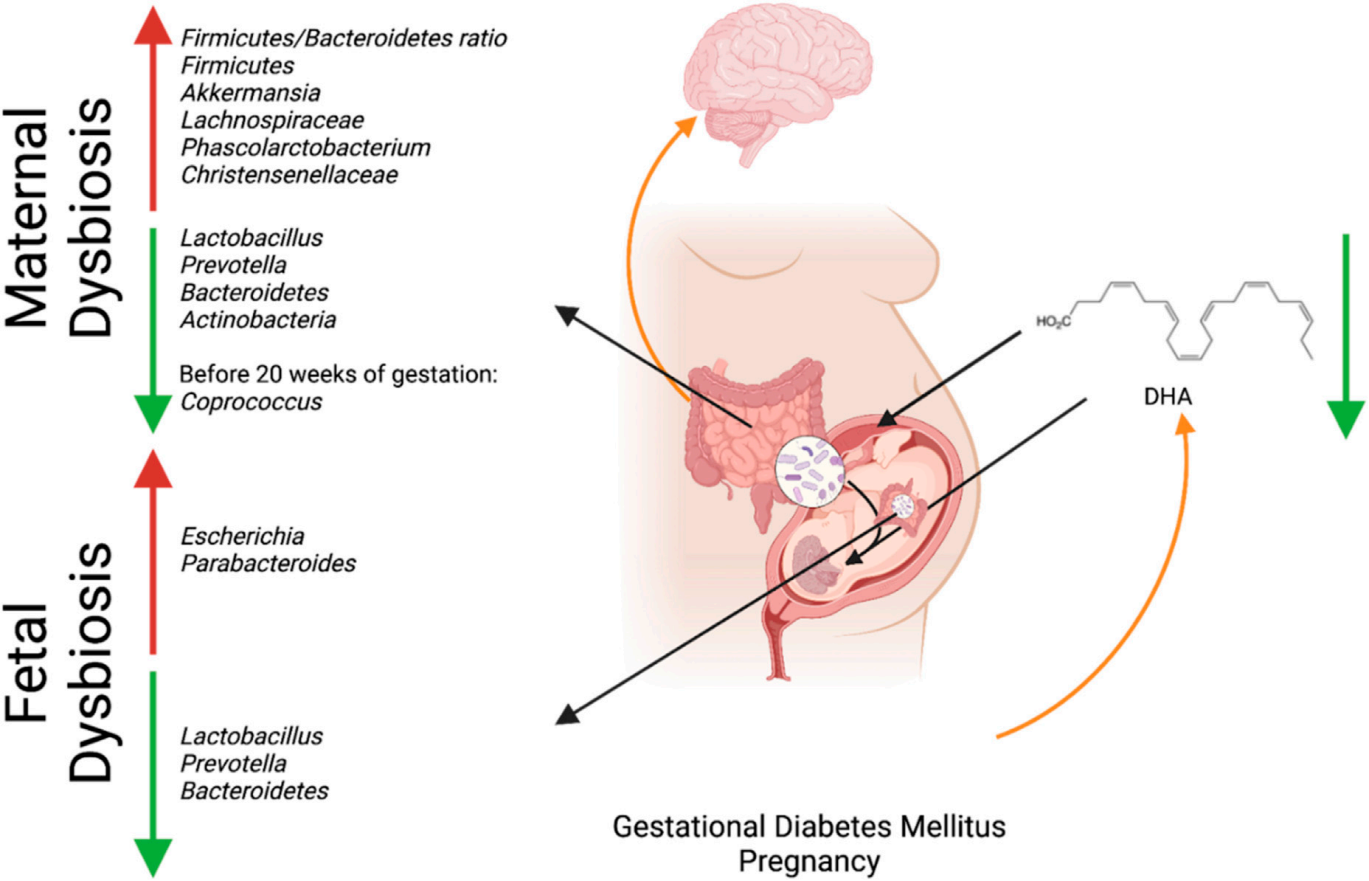
Interaction between maternal microbial dysbiosis and impaired maternal-fetal *in utero* translocation due to GDM and its connection to fetal neurodevelopment. Created with BioRender.com.

GDM has been associated with dysbiosis in the maternal microbiome and reduced availability of DHA in fetal circulation (Pagán et al., 2013; Neri et al., 2021). The dysbiosis in the maternal microbiome has led to an increase in SCFA-producing bacteria such as *Akkermansia* and a decrease in anti-inflammatory bacteria *Lactobacillus* and *Prevotella* (Eicher and Mohajeri, 2022). The increase in *Akkermansia* and decrease in the anti-inflammatory bacteria may have deleterious effects on fetal brain development, as maintaining physiologically relevant levels of SCFA and inflammation is crucial for decreasing the risk of psychiatric disorders. *Lactobacillus* and *Prevotella* play an essential role in early brain development: *Prevotella* produces vitamin B1 and modulates dopamine and serotonin expression for early brain development and *Lactobacillus* produces the neurotransmitter GABA, which has been connected to many neurodevelopmental diseases when deficient, as previously discussed (Sambon et al., 2021; Eicher and Mohajeri, 2022). The lack of DHA due to GDM may further promote dysbiosis in the fetal microbiome, as DHA helps maintain the microbiome’s *Firmicutes/Bacteroidetes* ratio and promotes the abundance of anti-inflammatory species (Muley and Plessis, 2018). As a result, providing high doses of DHA may increase the amount of DHA in the cord blood of GDM patients (Jiang et al., 2023a). Furthermore, GDM, the associated dysbiosis of the maternal gut microbiota, and the resulting impacts on metabolite delivery to the fetus should be studied. The effect of GDM on the maternal gut microbiome may restrict the production and transference of metabolites essential for fetal neurological and gut development (Figure 1; Muley and Plessis, 2018).

These findings are consistent with the studies analyzing the meconium microbiome of newborns born to mothers with GDM, which showed altered microbial species compared to newborns born to healthy mothers such as *Prevotella* (Kang et al., 2013; Jiang et al., 2015; Ponzo et al., 2019; Dicks et al., 2021; Li et al., 2021), *Bacteroides* (Jiang et al., 2015; Ponzo et al., 2019; Dicks et al., 2021; Li et al., 2021), and *Lactobacillus* (Aizawa et al., 2016; Ponzo et al., 2019; Li et al., 2021). Two studies found that GDM infants had a lower alpha diversity than normal control groups, showing that the mean species diversity in the GDM groups was different from that in the control groups (Su et al., 2018; Ponzo et al., 2019). The decrease in the presence of *Prevotella*, *Bacteroides*, and *Lactobacillus* has also been associated with anxiety and depression (Dicks et al., 2021), with *Prevotella* also being associated with the gastrointestinal disease associated with autism (Kang et al., 2013). Additionally, it was found that there was a significantly higher relative abundance of pro-inflammatory taxa such as *Escherichia* (Ponzo et al., 2019; Roth et al., 2021) and *Parabacteroides* (Jiang et al., 2015; Janssens et al., 2018; Ponzo et al., 2019; Wishart et al., 2022) in infants born to women with GDM than in infants born to healthy women. Altered levels of *Escherichia* and *Parabacteroides* could be a marker for potential future mental health issues, as *Parabacteroides* are significantly increased in a major depressive disorder group compared to healthy controls (Jiang et al., 2015), and *Escherichia* is a biomarker of depression in children as it is associated with producing serotonin (Roth et al., 2021; Andrioaie et al., 2022).

## Role of systems medicine paving ways for personalized treatment in GDM

Systems medicine is an interdisciplinary field of study that uses computational sciences and omic technologies to help solve biomedical problems. Integrating omics data across different layers—genomics, transcriptomics, proteomics, metabolomics, and microbiomics—offers a comprehensive view of the molecular landscape underlying GDM. Natalia et al. conducted the largest and the most ancestrally diverse genome-wide association meta-analysis study aimed at elucidating the close relationship between the pathobiology of type 2 diabetes (T2D) and GDM (Pervjakova et al., 2022). They identified five loci with significant genome-wide association, with GDM mapping to genes *MTNR1B*, *TCF7L2*, *CDKAL1*, *CDKN2A*-*CDKN2B*, and *HKDC1*, and single nucleotide polymorphisms (SNP) in them (snp ids: rs10830963, rs7903146, rs9348441, and rs10811662). In addition, they show, via Mendelian randomization analyses, a higher body mass index as a risk factor for GDM (Pervjakova et al., 2022). He et al. (2024) showed a correlation between gene polymorphisms in TCF7L2 and CAPN10 in GDM. Specifically, He et al. (2024) showed a strong correlation between SNV rs7903146 and the onset of GDM. Chermon and Birk (2024) showed an association of BDNF polymorphism with GDM and neurodevelopment. These studies pave the way for a precision medicine approach by screening for family history of T2D, higher BMI during pregnancy, and genotyping for SNVs in the susceptibility genes like rs7903146. Proteomics studies have shown placental abnormalities in women with GDM, with at least 37 proteins differentially upregulated compared to normal. Ten of these proteins are involved in fetal growth and development (Assi et al., 2020). If studies like these are established on a larger population, effective inhibitors of the pathways can be developed. A similar study using metabolomics has shown the identification of 212 metabolites showing significant differences in GDM placentas (Jiang et al, 2023b). Integromics approaches that combine genomics, transcriptomics, proteomics, and metabolomics data can be designed using machine learning tools to predict the risk of GDM among pregnant women. From here, a personalized treatment plan can be made for the patient to decrease the chances of developing GDM or to mitigate the impact of GDM through dietary modifications, exercise, supplementation of prebiotics, and the potential prescription of bacterial metabolism-targeted drugs (Hasain et al., 2020; Miko et al., 2022; Singh et al., 2023). Pinto et al. (2023) showed that microbiota-induced inflammation was identifiable months before gestational diabetes. Further studying the interaction of the maternal microbiome and microbial metabolites with the maternal-fetal gut microbiota and the fetal gut–brain axis can provide insights into the effects of dysbiosis in GDM patients on fetal neurodevelopment (Miko et al., 2022). Databases such as the Human Microbial Metabolome Database (MiMeDB) and Disbiome have started to build platforms for research workers to share their microbial species and microbial metabolite findings associated with certain diseases (Janssens et al., 2018; Wishart et al., 2022). These data can then be paired with metabolomics pathway analyzers such as MetaboAnalyst to identify metabolic abnormalities (Lu et al., 2022). In addition to metabolomics data, transcriptomics and genomic analysis should be collected from patients to identify protein and gene expression changes caused by or associated with the disease. With this overview of metabolomics, transcriptomics, genomics, and clinical data, biomarkers for the early detection of GDM and negative fetal neurodevelopment can be identified to reduce the harm of this complex condition.

By combining high-throughput quantitative molecular data used to identify the microbes and metabolites with different types of clinical information, possible metabolic deficiencies can be identified to improve and support fetal neurodevelopment in GDM pregnancies (Comte et al., 2020).

## Final thoughts and future directions

Further developing existing resources such as the Human Microbial Metabolome Database (MiMeDB) and Disbiome can allow the genes, microbes, and microbial metabolites associated with GDM to be cross-referenced across different studies and analyzed to determine which maternal microbes are affected by GDM and the most accurate techniques for consistently identifying these species (Janssens et al., 2018; Wishart et al., 2022). Additionally, the transportation of the abundant or deficient metabolites from the mother to the fetus can be studied to determine the downstream effects on fetal neurodevelopment. Utilizing a systems medicine approach, this information can be used to design personalized treatment plans for patients based on the abundance levels of these microbes and microbial metabolites in the maternal gut microbiome and cord blood to supplement the nutrients needed for fetal neurodevelopment in GDM pregnancies. Accumulating data on the roles of pro-inflammatory cytokines in the initiation of GDM ML models can be built to predict which patients may develop GDM to allow for preventative measures and reduce the deleterious effects on fetal development.
